# Operator-Driven Active Craniospinal Tensioning (ACT) Device for Non-ambulatory Application

**DOI:** 10.7759/cureus.114854

**Published:** 2026-08-20

**Authors:** Huan-Wei Chen

**Affiliations:** 1 Chiropractic, Private Chiropractic Practice, Vancouver, CAN

**Keywords:** active craniospinal tensioning (act), alzheimer's disease, amyotrophic lateral sclerosis (als), cerebral venous preconditioning (cvpc), craniocervical junction, dural pull-recoil, glymphatic system, pelvis-stabilized axial spinal traction (psast), remote ischemic preconditioning (ripc)

## Abstract

Active craniospinal tensioning (ACT) is an upright-posture axial spinal traction maneuver hypothesized to couple two mechanisms: dural pull-recoil, a cerebrospinal fluid (CSF) pressure-gradient effect shared with the supine technique pelvis-stabilized axial spinal traction (PSAST), and suboccipital venous occlusion-rebound, proposed to promote central nervous system (CNS) venous and lymphatic drainage, and as a distinct modality for cerebral venous preconditioning (CVPC). The CVPC mechanism depends on upright posture. In the upright position, cerebral venous return preferentially routes through the suboccipital venous plexus rather than the internal jugular veins, allowing the ACT strap to transiently occlude this outflow pathway during the hold and produce a hemodynamic rebound upon release. This CVPC-based rationale for choosing ACT over PSAST, along with the full biomechanical model, was developed in prior papers and is not re-derived here.

In its originally described form, ACT is participant-driven: the participant voluntarily squats against a fixed overhead strap secured by a simple anchor, and the participant's own hands close the strap loop as a fail-safe. This report refers to that form, and the strap-and-anchor apparatus that delivers it, as active craniospinal tensioning, self-administered (ACT-SA). ACT-SA's delivered craniocervical junction (CCJ) tension depends on the participant fully relaxing into the maneuver. It categorically excludes non-ambulatory participants with advanced neurodegenerative disease, e.g., Alzheimer's disease (AD) and amyotrophic lateral sclerosis (ALS), who cannot generate or safely control a voluntary squat, yet who may stand to benefit most from craniospinal tensioning and CVPC given their disease-associated glymphatic impairment.

This report describes active craniospinal tensioning, operator-driven (ACT-OD), a device that reproduces ACT-SA's anchor geometry, target force range, and hold duration while substituting the source of tensioning force. Rather than gravity acting on a squatting participant's own body mass, tension is generated by a practitioner operating a pulley-and-lever mechanism against a stationary, seated participant, with the strap confined near the forehead by a polyvinyl chloride (PVC) pipe rather than closed by the participant's own hands. Because the participant does nothing and the seated default state carries no CCJ tension, the fail-safe ACT-SA requirement becomes unnecessary; disengagement, not engagement, is ACT-OD's default. Freed from managing the strap, the practitioner can devote full attention to the lever and to continuous observation of the participant's response, a substitution that matters because non-verbal or cognitively impaired participants cannot reliably self-report the presyncopal symptoms that serve as ACT-SA's real-time safety signal.

The design is offered without patent restrictions for research and clinical replication. This report presents device design and mechanism only, with no clinical outcome or efficacy data.

## Introduction

The glymphatic system is a perivascular clearance pathway increasingly implicated in the accumulation of metabolic and proteinaceous waste associated with neurodegenerative diseases, including amyloid-beta and tau in Alzheimer's disease (AD), as well as misfolded protein aggregates in amyotrophic lateral sclerosis (ALS) [1]. Axial spinal traction has been proposed as a candidate biomechanical means of modulating cerebrospinal fluid (CSF) and glymphatic circulation, first in a supine, practitioner-applied form, pelvis-stabilized axial spinal traction (PSAST) [2], and subsequently in an upright, participant-driven form delivered via a simple strap-and-anchor apparatus, originally described simply as active craniospinal tensioning (ACT) [3,4] and termed here active craniospinal tensioning, self-administered (ACT-SA) to distinguish it from the operator-driven variant introduced in this report.

Both techniques share a dural pull-recoil mechanism: axial elongation transiently tensions the spinal dural sac and generates a CSF pressure gradient across the foramen magnum that is reversed on release [2-4]. PSAST, however, is performed supine, a posture in which cerebral venous return is carried predominantly by the internal jugular veins, and suboccipital venous occlusion during the maneuver is not appreciable [2]. Active craniospinal tensioning (ACT) is performed upright, a posture in which cerebral venous outflow shifts preferentially toward the suboccipital venous plexus [5,6]. Because the ACT strap crosses this plexus at the suboccipital contact point, the same upright tensioning maneuver produces transient suboccipital venous occlusion-rebound, proposed as a distinct modality for cerebral venous preconditioning (CVPC), a venous-side analog to remote ischemic preconditioning (RIPC) [7]. The full CVPC mechanism, safety parameters, and falsifiable diffusion tensor image analysis along the perivascular space (DTI-ALPS) endpoint are developed in the prior ACT/CVPC report [4] and treated here as established background rather than re-derived. The present report is concerned solely with extending ACT's delivery to participants who cannot generate it themselves.

ACT-SA generates axial tension entirely through a voluntary squat: the participant transfers partial body weight through the spine via the suboccipital contact area to a fixed overhead strap, and the participant's own hands close the strap loop as a fail-safe that releases automatically if grip is lost [3,4]. This design has two consequences relevant here. First, delivered craniocervical junction (CCJ) tension is behaviorally constrained; prior self-observation indicates that incomplete relaxation, with residual lower-limb activity partially supporting body weight through the floor, reduces the load actually transmitted to the suboccipital anchor, so the participant's effort and relaxation state, not just body weight, determine the outcome [4]. Second, and more categorically, ACT-SA requires the participant to be able to stand, to squat actively, and to maintain a voluntary grip throughout, capacities that are absent in an appreciable fraction of participants with advanced AD or ALS, precisely the population in which glymphatic clearance is most compromised.

This report describes active craniospinal tensioning, operator-driven (ACT-OD). This device closes this gap by substituting the source of tensioning force while preserving ACT-SA's anchor geometry and target force range. The report is organized around a direct comparison between ACT-OD and ACT-SA, since ACT-OD is best understood not as a new intervention but as a tensioning-source variant of an already described ACT. The objective of this report is therefore narrow and specific: to describe ACT-OD's mechanical design, force-delivery parameters, and operational safety framework, and to establish why the ACT/CVPC mechanistic rationale developed previously for ACT-SA [4] applies to ACT-OD without modification. This is a device-design and biomechanical-rationale report; it does not present clinical outcome, efficacy, or physiological-equivalence data, and no such claims should be inferred from the mechanical description that follows.

## Technical report

Device overview

The participant sits in a chair facing a fixed vertical post and is secured by a trunk safety belt. The belt itself is not a specified component; any commercially available strap, harness, or belt of adequate strength and width to secure the trunk snugly is sufficient. A 48-50 mm nylon or polyester strap, identical to the ACT-SA strap [3,4], is routed from beneath the external occipital protuberance, forward and superiorly over the lower ear, and confined 5-10 cm in front of the forehead by a 1-inch-diameter, approximately 4-inch-long polyvinyl chloride (PVC) pipe. The pipe length was chosen specifically because a 4-inch section is easy for the practitioner to grip and position one-handed while confining the strap. The strap passes through the interior of the PVC pipe, which holds the loop together by confinement alone once positioned appropriately; no further manual holding is required, allowing the practitioner to direct full attention to operating the lever and to observing the participant during the procedure. The confined strap connects to a rope that runs up over the pulleys at the top of the post and down the opposite side to a lever. Pushing the lever down draws the strap upward, generating axial tension through the suboccipital contact against the participant's sitting weight. The practitioner lifts until the participant's hips clear the chair, confirmed tactilely and visually, at which point CCJ tension is maximized at sitting weight minus estimated head weight (~9-12 lb) and does not increase further with additional lift height. The lift-hold-release sequence follows the same hold-duration parameters as ACT-SA and PSAST: a standard 5-second hold (matching the upper bound of ACT-SA's and PSAST's typical range), extendable to a strict 10-second maximum at practitioner discretion, contingent on continuous monitoring for non-verbal distress since self-reported presyncopal symptoms may be unavailable in this population. Delivered force is approximately 60-70% of body weight at full relaxation, distinct from PSAST's practitioner-adjustable 50-80% range, as detailed in the Discussion section. As with ACT-SA and PSAST, ACT-OD is intended for administration at one to three sessions per day, consistent with the dosing established in the prior ACT/CVPC framework [3,4].

**Figure 1:**
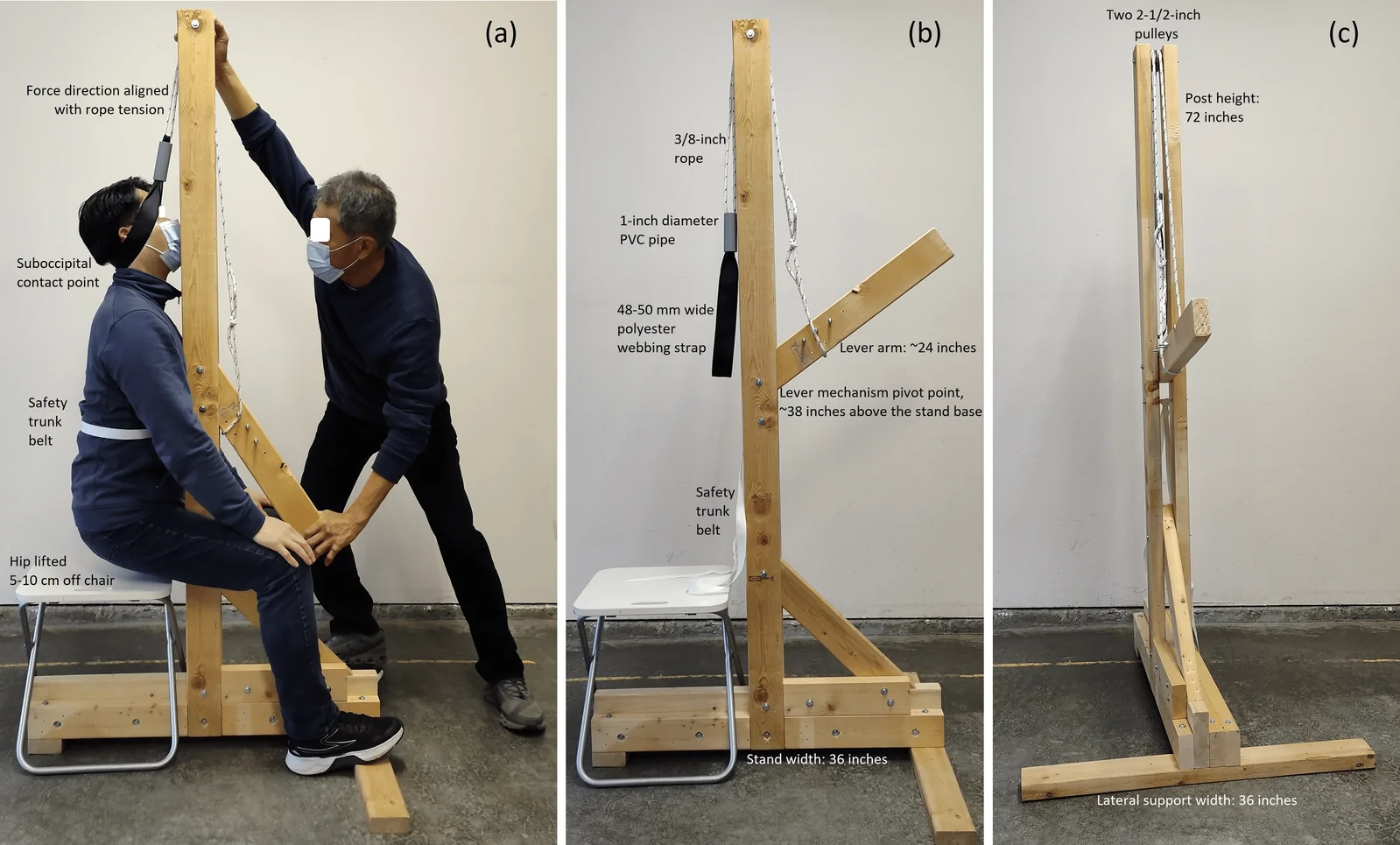
Active craniospinal tensioning, operator-driven (ACT-OD): device demonstration (a) The participant is seated in a chair facing the device's vertical post and is secured with a safety belt around the trunk to prevent falls. A 48-50 mm wide nylon or polyester webbing strap is routed from beneath the external occipital protuberance anteriorly and superiorly, passing over the lower ear, primarily the earlobe, and confined 5-10 cm in front of the forehead by a polyvinyl chloride (PVC) pipe, extending upward through a rope-and-pulley mechanism to the other side of the post, and ending at the lever. This confinement allows the practitioner to secure the strap without holding it manually throughout the procedure. Sitting weight is measured prior to the procedure; maximum deliverable craniocervical junction (CCJ) tension is calculated as measured sitting weight minus estimated head weight (~9-12 lb). The practitioner pushes down on the lever over approximately 2 seconds, drawing the strap upward via the rope-and-pulley mechanism and lifting the participant 5-10 cm clear of the chair, sufficient to fully unweight the seated position. This position is held for approximately 5 seconds, the standard duration, extendable to a strict maximum of 10 seconds at the practitioner's discretion, with continuous monitoring for non-verbal distress. The practitioner then releases force on the lever over approximately 1 second, lowering the participant back onto the chair. (b) Side view of the ACT-OD device. (c) Front view of the ACT-OD device. Image credit: Photograph by author

The complete device consists of a post-and-stand, pulleys, lever, strap, rope, PVC pipe, and safety trunk belt, constructed entirely from standard hardware-store components with no specialized fabrication or proprietary parts (Figure 1; Video 1; Table 1). The post height and pulley positions are fixed; accommodation for participants of different stature is instead achieved through adjustable rope length and a multi-setting lever, matching the tensioning geometry to the participant's sitting height without altering the post itself. The chair is not a fixed component of the device: any chair of suitable height may be substituted at the practitioner's discretion, provided it allows the trunk belt to be secured snugly and positions the participant facing the post with the suboccipital contact point reachable by the strap. The device is designed to accommodate a standard non-motorized wheelchair in place of a chair, though this configuration has not been validated separately and is offered as design intent rather than a tested specification.

**Video 1:** Active craniospinal tensioning, operator-driven (ACT-OD): device demonstration This brief video demonstrates one operational session of an ACT-OD device, developed for research purposes to deliver controlled craniocervical junction (CCJ) tension in a seated position. The clip shows: (1) the operator engaging the lever to draw the strap upward via the pulley-and-lever mechanism, (2) the participant being lifted 5-10 cm clear of the chair, fully unweighting the seated position, held for the standard 5-second duration, and (3) controlled release, lowering the participant back to the chair. Both individuals depicted are consenting volunteers; no patient is depicted. A caption has been added to describe the sequence shown. This footage accompanies a technical report describing the ACT-OD device design, safety parameters, and proposed applications that was submitted to Cureus. It is provided for illustrative and reproducibility purposes only and does not constitute medical advice. The device is intended for use by trained practitioners only.

**Table 1:** Materials list for active craniospinal tensioning, operator-driven (ACT-OD) prototype construction Bolt use and placement, including the pulley axle and lateral-support joints, are shown in Figure 1. Where a bolt is too short for the nut and washer to seat flush at the outer face, a counterbore recess accommodates the nut/washer stack. A 0.5-inch recess depth is used, leaving approximately 1 inch of solid lumber (based on 1.5-inch actual 2-by lumber thickness) around the recess to preserve structural integrity. Two PET (polyethylene terephthalate) plastic spacers are placed on each side of the lever arm's pivot contact, lubricated with petroleum jelly, to provide smooth, low-friction, noise-free operation at this interface. In the device's double-pulley (2:1) configuration, the rope's manufacturer-rated capacity of ~150 lb per strand yields a system lifting capacity of approximately 300 lb, a safety margin of over 2-fold above the estimated working load (~130 lb) required for this application.

Component	Specification	Cut length/size	Quantity
Lumber, 2×4	Nominal 2×4, actual 1.5×3.5 inch	72 inches	2
Lumber, 2×4	Nominal 2×4, actual 1.5×3.5 inch	36 inches	2
Lumber, 2×4	Nominal 2×4, actual 1.5×3.5 inch	16.25 inches	4
Lumber, 2×4	Nominal 2×4, actual 1.5×3.5 inch	25 inches (includes ~1.5-inch reserve beyond pivot bolt for structural integrity; effective lever arm ~24 inches)	1
Lumber, 2×4	Nominal 2×4, actual 1.5×3.5 inch	13 inches	1
Lumber, 2×4	Nominal 2×4, actual 1.5×3.5 inch	23 inches, 45-degree cut both ends	1
Lumber, 2×6	Nominal 2×6, actual 1.5×5.5 inch	36 inches	1
Hex bolt (UNC), Grade 5, zinc-plated	1/4×3.5-inch	—	6
Hex bolt (UNC), Grade 5, zinc-plated	1/4×5-inch	—	8
Hex bolt (UNC), Grade 5, zinc-plated	1/4×6-inch	—	5
Nut, 1/4-inch UNC	—	—	19
Washer, 1/4-inch	—	—	38
PVC pipe, Schedule 40 DWV	1-inch diameter	4 inches	1
Pulley, single-sheave fixed-eye, 551-lb safe working load	2.5-inch, max 3/8-inch rope	—	2
Poly rope, braided, all-purpose	3/8-inch, ~150 lb rated capacity	cut to size	1
PET plastic spacer	repurposed food-container plastic	cut to fit	4
Polyester webbing strap	heavy-duty, 48–50 mm wide	40 inches, sewn into loop	1

Safety and operational control

Force and duration are controlled directly by the practitioner throughout the procedure. ACT-OD does not require a manual-holding fail-safe built into the ACT-SA strap design [3,4] because the participant's default seated position already results in no force applied to the CCJ, and disengagement (rather than continued tension) is the default outcome if the practitioner releases the lever for any reason. The participant's trunk remains independently secured to the post by a separate safety belt throughout, ensuring the participant is supported against a fall regardless of the tensioning strap's condition. The ACT-OD device is intended solely for administration by a trained practitioner and is not designed for unsupervised use or operation by untrained individuals.

## Discussion

The following case uses the author's own measured values as the participant depicted in Figure 1 and Video 1, illustrating the force-margin methodology used throughout this Discussion rather than constituting a study sample: body weight 175 lb, measured sitting weight 130 lb, estimated head weight ~11 lb, yielding an estimated maximum CCJ tension of ~119 lb, or ~68% of body weight.

ACT-OD as a tensioning-source variant, not a new mechanism

ACT-OD is designed to be understood as a tensioning-source variant of the already-described ACT mechanism rather than as an independent intervention requiring separate mechanistic justification. The anchor geometry and suboccipital contact point are unchanged from ACT-SA [3,4]; only the source of the applied force changes, from gravity acting on a voluntarily squatting participant to a practitioner operating a lever against a stationary one. Because the upright posture and the suboccipital contact point are preserved unchanged, the CVPC rationale developed for ACT generally, that upright posture routes cerebral venous return through the suboccipital venous plexus, making transient occlusion-rebound of that outflow possible in a way it is not in the supine PSAST posture [2,4], applies to ACT-OD without modification. The 50-80% body-weight force range established for PSAST [2], consistent with the author's clinical practice, reflects a practitioner-selected target that is achievable because the PSAST force is applied directly and can be adjusted continuously per patient. ACT-SA and ACT-OD, by contrast, generate tension from body weight transmitted through the suboccipital contact point under gravity (ACT-SA) or an equivalent lift (ACT-OD), rather than from directly applied practitioner force. In both techniques, head weight and a portion of lower-extremity weight remain anatomically excluded from this pathway; in ACT-SA, weight borne through the hands' grip on the strap is excluded as well, further reducing delivered tension by an estimated 5-8% relative to ACT-OD, based on the author's experience with both techniques. Given the case above (~68% for ACT-OD), full relaxation in ACT-SA and ACT-OD is therefore expected to yield a force of approximately 60-70% of body weight, below PSAST's upper bound. Sitting, in contrast to squatting, is a naturally relaxed posture, allowing ACT-OD's practitioner-controlled lift to reliably reach this same anatomically determined value at full unweighting, without the participant-effort-dependent variability that affects ACT-SA. The dural pull-recoil mechanism underlying ACT's CSF pressure-gradient and glymphatic-clearance rationale therefore applies to ACT-OD without modification. This report does not re-derive that mechanism; it extends its delivery to a population that could not previously receive it.

Whether mechanical equivalence implies physiological equivalence

Reproducing ACT's mechanical input without participant effort does not resolve whether practitioner-delivered tensioning produces the same physiological effect (dural pull-recoil, suboccipital venous occlusion-rebound, and any downstream glymphatic or CVPC consequence) as participant-delivered tensioning. This comparison remains hypothetical on both sides: ACT-SA's physiological effects have not been empirically confirmed, so ACT-OD's equivalence to ACT-SA is a comparison between two unvalidated mechanisms rather than a validation against an established baseline. If anything, the removal of participant-effort-dependent variability in ACT-SA [3,4] suggests that ACT-OD may deliver more consistent tension than ACT-SA, but this remains a mechanical inference rather than an empirical finding. Dedicated validation, potentially using DTI-ALPS [8,9] as an objective endpoint consistent with the falsifiable approach already proposed for ACT generally [3,4], is required before any claim of physiological or clinical equivalence can be made for either technique.

Duration, monitoring, and the limits of fixed-duration safety

The standard 5-second hold is inherited from the self-observation underlying ACT-SA [3,4]. Throughout the hold, from its start, the practitioner monitors the participant continuously and terminates the maneuver immediately upon any sign of distress, whether verbally reported (in participants able to communicate) or observed non-verbally (facial grimace, altered respiratory pattern, or other visible signs) [10] in participants who cannot reliably self-report. This continuous monitoring requirement applies throughout the entire hold, not only beyond the standard duration. Some degree of discomfort is expected and anticipated at the standard 5-second duration; it is a distress signal, not discomfort itself, that triggers immediate termination. If no distress signal is observed within 5 seconds, the practitioner may, at their discretion, extend the hold to a strict 10-second maximum, provided monitoring continues uninterrupted. Both durations were chosen in round, 5-second increments for operational simplicity rather than derived from a precisely identified physiological threshold. Based on the author's personal experience administering ACT-SA, discomfort escalates progressively over this window, becoming markedly more pronounced by 10 seconds, but, from that same experience, this duration is judged to remain within a tolerable range, well short of syncope. Ten seconds, therefore, represents a deliberately conservative ceiling on acceptable discomfort rather than an empirically derived edge of physiological risk, and it is an absolute maximum: the maneuver must be terminated at or before this point, regardless of the participant's observed status. Because this judgment is based on a single practitioner's self-experienced tolerance, its generalizability to the ACT-OD population, who may differ in age, disease state, and pain tolerance, is unconfirmed and is identified as a limitation. A further limitation is that non-verbal distress monitoring, on which extension decisions rely for participants unable to self-report, has not been validated against the self-report signal it substitutes for in ACT-SA. The relative sensitivity of non-verbal monitoring compared to self-report is unknown.

Trigeminal considerations

Beyond the primary suboccipital contact, the strap crosses the lower ear, mandibular, and maxillary regions, engaging upper cervical/great auricular (C2-C3) and trigeminal (V2/V3) territories. Mechanical stimulation in the trigeminal territory is a recognized trigger for the trigeminocardiac reflex (TCR) [11]. Still, the TCR literature describes this reflex arising primarily from direct, sustained, or repeated intraoperative manipulation of the trigeminal nerve or ganglion, a substantially different exposure from the brief external compression described here (~5 seconds, up to a 10-second maximum). A clinically significant TCR response is considered unlikely under standard administration and is noted as a minor contributing consideration. Presyncopal symptoms under this maneuver are considered more likely attributable to the intended suboccipital venous occlusion-rebound and CVPC mechanism than to TCR.

Force margins

This section revisits the case introduced at the start of the Discussion (body weight 175 lb; measured sitting weight 130 lb; estimated head weight ~11 lb; estimated maximum CCJ tension ~119 lb, 68% of body weight) to illustrate the force-margin methodology, using the same participant depicted in Figure 1 and Video 1, rather than constituting a study sample. This figure is a calculated estimate rather than a direct force measurement: sitting weight is measured per participant, but head weight is a standard anthropometric approximation rather than participant-specific, and no load cell or other direct instrumentation was used to confirm delivered CCJ tension. Notably, even direct instrumented force measurement (e.g., an inline load cell) would not eliminate this estimation step: such a measurement would capture total force delivered through the strap, from which head weight would still need to be deducted to isolate CCJ-specific tension, leaving the same anthropometric approximation (~11 lb in this case) necessary regardless of measurement method. Against cadaveric tensile failure thresholds of ~1,555 N (skull-T3 preparations) and ~3,373 N (fully intact cadavers) [12], this estimated tension (119 lb=529 N) represents safety margins of ~2.9-fold and ~6.4-fold, respectively. Two caveats apply, both of which likely make these margins conservative underestimates rather than overestimates: the cited cadaveric data were obtained under dynamic (1 m/s) loading on a small number of specimens of unreported demographic characteristics, whereas ACT-OD applies force quasi-statically over several seconds, and passive cadaveric tissue, even where musculature is anatomically intact, lacks the active muscular resistance and reflexive guarding that living cervical tissue would contribute under equivalent loading. Because specimen age, bone density, and degenerative status are unreported, applicability to elderly participants with neurodegenerative disease, a population more likely to present with osteoporosis or connective tissue changes, cannot be assumed from the cadaveric data alone.

These calculated safety margins are presented for illustrative purposes only and do not, by themselves, establish clinical device safety. Direct instrumented force measurement, including calibration, measurement uncertainty, and repeatability testing, together with formal safety validation in the intended participant population, is required before any safety claim can be made. Throughout this report, measured observations, calculated estimates, and hypothesized mechanisms or outcomes are kept distinct; no clinical or physiological outcome was measured; and claims regarding CSF pressure, glymphatic clearance, CVPC, or clinical benefit remain explicitly hypothesized rather than demonstrated.

Contraindications, precautions, and disclaimer

Comprehensive contraindications, precautions, and general safety considerations associated with axial spinal traction have been described previously for PSAST [2] and remain applicable here; they are not repeated in full but should be considered integral to the application of this device. Given ACT-OD's capacity to fully unweight the seated position and deliver tension deterministically rather than behaviorally, particular attention should be given to bone density and cervical spine integrity.

Unlike ACT-SA [3,4], which is driven by the participant's own voluntary squat and depends on the participant's physical and cognitive capacity to terminate the maneuver, ACT-OD is entirely operator-driven: force, duration, and termination are governed at all times by the operating practitioner. This device is not intended for suspension, prolonged loading, or any activity that may pose a risk of self-injury. Deviation from the described protocol, inappropriate application, or use by untrained individuals may result in serious injury.

Because the participant does not generate or control the maneuver, ACT-OD is not intended for self-administration or lay operation under any circumstances, and requires administration by a practitioner trained in axial spinal traction technique. The mandatory presence of a trained operating practitioner, together with the trunk-securing safety belt, serves as the safeguard analogous to the safety spotter required for ACT-SA [3,4]. The author assumes no responsibility for adverse outcomes arising from use outside the conditions, parameters, and professional oversight described in this report.

This report describes device design and biomechanical rationale only, illustrated by a mechanical demonstration involving two consenting volunteers; it does not report clinical outcomes, efficacy data, or a human trial. ACT-OD is offered as a research-use-only prototype; clinical application requires appropriate ethics review and documented informed consent, consistent with local regulatory requirements.

## Conclusions

This report describes ACT-OD, which extends the previously described ACT framework (termed ACT-SA in this report) and, through it, the ACT/CVPC mechanism established in prior work, to non-ambulatory participants by substituting the source of tensioning force. While ACT-SA generates tension through a participant's voluntary squat against a fixed strap, requiring the participant to close the strap loop as a fail-safe, ACT-OD generates tension through a practitioner-operated lever against a stationary, seated participant, whose strap is instead mechanically confined by a PVC pipe. Because tension exists only while the practitioner actively works the lever, disengagement is the system's default state, eliminating the need for ACT-SA's fail-safe. Because the participant need not hold or grip anything, the seated posture is more natural, and the delivered tension is expected to be more consistent than ACT-SA's participant-effort-dependent delivery. Freed from strap management, the practitioner can devote full attention to the lever and continuous non-verbal monitoring, which is intended to substitute for the self-reported presyncopal symptoms that may be unavailable in this population.

This report demonstrates mechanical feasibility only. The calculated CCJ tension values and cadaveric-based safety margins presented in this report are mechanical estimates, not direct force measurements or evidence of established clinical safety. Similarly, any reference to CSF dynamics, glymphatic clearance, or cerebral venous preconditioning reflects a proposed mechanism carried forward from prior work, not a physiological or clinical outcome measured in this report or in ACT-SA. Formal safety, physiological-equivalence, and efficacy studies, potentially using DTI-ALPS as an objective endpoint, are required before any clinical application of ACT-OD or ACT-SA.
